# The International Hahnemannian Association

**Published:** 1897-07

**Authors:** 


### THE INTERNATIONAL HAHNEMANNIAN ASSOCIATION.

The eighteenth annual meeting of the International Hahnemannian Association was held in the International Hotel, at Niagara Falls, New York.

The first meeting was on Tuesday, June 29th, at 11 A. M., and was called to order by the President, Wm. P. Wesselhoeft, M. D., who then read an excellent and eloquent address that was truly homeopathic in every sense, and to the point for which the Association was formed. The President's address was strongly applauded by the members present.

The report of the Corresponding Secretary, Mary Florence Taft, was then read by Dr. Case. Dr. Taft was not present, owing to severe illness in her family. Dr. Taft reported having written to numbers of foreign members, and as a result two very valuable papers had been received. She quoted part of one from Eleanor L. Blonde, M. D., entitled, "Skin Diseases in the Hawaiian Islands." The remainder of this interesting paper was read at a later meeting under the report of Bureau of Clinical Medicine.

The Necrologist, S. T. Guild Leggett, M. D., sent her report, announcing the deaths of Dr. John Hall, Toronto, Canada; Dr. Alva Harvey, Springfield, Mass.; Dr. Stephen

A. Seward, Syracuse. The Secretary then read letters of resignation from Drs. G. H. Clark, Leslie Martin, and W. E. Ledyard. These resignations were not accepted. Drs. Ledyard and Martin were by a unanimous vote placed on the list of honorary Seniors, and the Secretary was requested to write to Dr. Clark, urging a reconsideration of his decision.

Two new members were proposed, Dr. Rita M. Langton for regular membership, and Dr. Jos. E. Fitzsimmons associate member; both were elected.

The Treasurer being absent, his report was deferred.

Morning, afternoon, and evening sessions were held, and full and interesting reports were read from the various bureaus.

The closing session was held Thursday, July 1st, at 3.30 P. M. Dr. Thomas M. Dillingham was elected President for the ensuing year. Dr. Alice B. Campbell Vice-President, Dr. Case Secretary, and Dr. Powell Treasurer.

Among the doctors present were: C. M. Boger, W. Va.; Dr. Annie Lowe Geddes, J. Henry Allen, James B. Bell, Thomas M. Dillingham, Henry W. Pierson, B. L. B. Baylies, D. C. McLaren, Allen B. Carr, S. F. Shannon, H. C. Allen, L. A. L. Day, T. Dwight Stow, Alice Campbell, Montague Leverson, W. M. James, and the venerable Dr. Arthur Fisher, from Montreal.

It was a feast of reason and a flow of soul. The meeting adjourned with the best feelings of fraternal brotherhood among its members, and those who were there were very glad they came. and those who did not have cause to regret, repent, and appear at next year's meeting.

---

THE place of meeting of the International Hahnemannian Association has not yet been chosen. It is proposed to make this meeting most interesting and profitable. Let all come —old members, new members, and former members. You won't regret *coming*.

